# Supplementary figures and images for: Multicenter evaluation of BACT-Info. and an infection algorithm using Urine Flow Cytometry among clinically diagnosed UTI patients in Indonesia
Source: PLoS One. 2026 Jul 15;21(7):e0339255. doi: 10.1371/journal.pone.0339255 (PMC13372243; doi:10.1371/journal.pone.0339255)

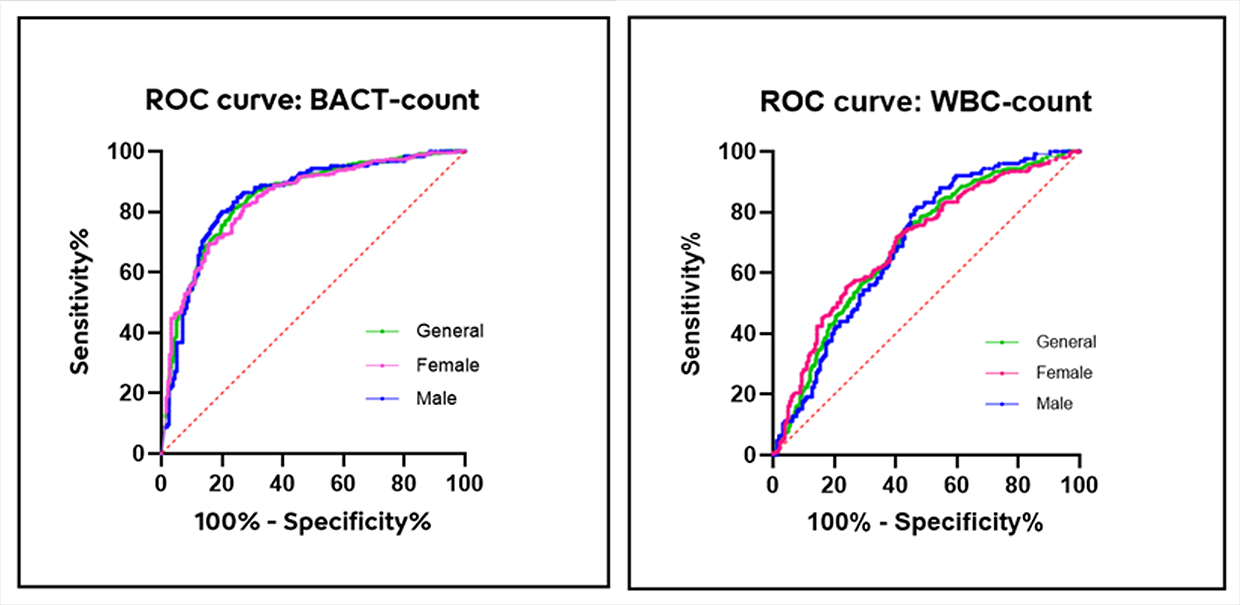

Supplement: S1 Fig — When patients were further categorized based on sex, for both BACT-count (left) and WBC-count (right), the curves for general, female and male populations largely overlap, indicating consistent diagnostic accuracy regardless of sex. However, the BACT-count consistently demonstrates superior performance compared to the WBC-count, with its curve positioned further from the line of no discrimination, suggesting it is a more accurate diagnostic marker. (TIF) [file pone.0339255.s001.tif]
