# Supplementary material for: Multicenter evaluation of BACT-Info. and an infection algorithm using Urine Flow Cytometry among clinically diagnosed UTI patients in Indonesia
Source: PLoS One. 2026 Jul 15;21(7):e0339255. doi: 10.1371/journal.pone.0339255 (PMC13372243; doi:10.1371/journal.pone.0339255)
Supplement: S1 Table — The most commonly isolated pathogen in this study was Escherichia coli, accounting for 37.41% of all isolates, followed by Klebsiella pneumoniae (11.76%) and Burkholderia cepacia (6.12%). Other notable bacteria included Enterococcus faecalis and Pseudomonas aeruginosa (each 5.88%), while fungal isolates such as Candida albicans (2.82%) and Candida tropicalis (1.88%) were also detected. Less frequent pathogens included Acinetobacter baumannii (2.35%), Staphylococcus epidermidis (2.12%), and Proteus mirabilis (1.88%). Additionally, 6.35% of isolates were classified as other Gram-positive bacteria, 11.53% as other Gram-negative bacteria, and 4.00% as other fungi. (DOCX) [file pone.0339255.s002.docx]

| Microorganism | n (%) |
| --- | --- |
| *Escherichia coli* | 159 (37.41) |
| *Klebsiella pneumoniae* | 50 (11.76) |
| *Burkholderia cepacia* | 26 (6.12) |
| *Enterococcus faecalis* | 25 (5.88) |
| *Pseudomonas aeruginosa* | 25 (5.88) |
| *Candida albicans* | 12 (2.82) |
| *Acinetobacter baumannii* | 10 (2.35) |
| *Staphylococcus epidermidis* | 9 (2.12) |
| *Candida tropicalis* | 8 (1.88) |
| *Proteus mirabilis* | 8 (1.88) |
| Other gram positive | 27 (6.35) |
| Other gram negative | 49 (11.53) |
| Other fungi | 17 (4.00) |
