# Supplementary material for: Multicenter evaluation of BACT-Info. and an infection algorithm using Urine Flow Cytometry among clinically diagnosed UTI patients in Indonesia
Source: PLoS One. 2026 Jul 15;21(7):e0339255. doi: 10.1371/journal.pone.0339255 (PMC13372243; doi:10.1371/journal.pone.0339255)
Supplement: S2 Table — For the general population, a BACT-count cutoff of 975.4/µL yielded 81.3% sensitivity and 76.3% specificity. Among males, the optimal BACT-count cutoff was 1008/µL (80.0% sensitivity, 80.1% specificity), while for females it was 967.7/µL (81.9% sensitivity, 72.9% specificity). For WBC-count, the general population cutoff was 82.05/µL with 72.7% sensitivity and 58.5% specificity. In males, a lower cutoff of 51.4/µL achieved higher sensitivity (81.6%) but moderate specificity (53.2%), whereas in females, a cutoff of 83.0/µL yielded 71.8% sensitivity and 59.7% specificity. These findings suggest good diagnostic potential of UF parameters, particularly BACT-count, across different subgroups. (DOCX) [file pone.0339255.s003.docx]

| **Parameter** | **Optimal Cutoff (/µL)** | **Performance** | |
| --- | --- | --- | --- |
|  |  | **Sensitivity (%)** | **Specificity (%)** |
| **BACT-count** |  |  |  |
| General population | 975.40 | 81.3 | 76.3 |
| Male | 1008.00 | 80.0 | 80.1 |
| Female | 967.70 | 81.9 | 72.9 |
| **WBC-count** |  |  |  |
| General population | 82.05 | 72.7 | 58.5 |
| Male | 51.40 | 81.6 | 53.21 |
| Female | 83.00 | 71.8 | 59.7 |
