# Supplementary material for: Multicenter evaluation of BACT-Info. and an infection algorithm using Urine Flow Cytometry among clinically diagnosed UTI patients in Indonesia
Source: PLoS One. 2026 Jul 15;21(7):e0339255. doi: 10.1371/journal.pone.0339255 (PMC13372243; doi:10.1371/journal.pone.0339255)
Supplement: S3 Table — UF-5000/4000 Flag Results Based on Gram Type of Urine Culture Results. Among the 721 total samples, urine culture identified 318 as Gram-negative, 59 as Gram-positive, 7 as mixed bacterial, and 337 as sterile. The UF system flagged 118 samples as Gram-negative, of which 104 matched the culture results. It flagged 172 as Gram-positive, with only 40 correctly identified. The mixed flag was applied to 143 cases, though only 5 were confirmed as mixed. A total of 199 samples showed no flag, most of which (168) were truly sterile in urine culture. This distribution suggests that while the UF system has a relatively good ability to flag Gram-negative bacteria, its accuracy is lower for detecting Gram-positive and mixed-Gram bacterial UTIs. (DOCX) [file pone.0339255.s004.docx]

| **UF BACT-Info. Flag** | **Bacterial Culture** | | | | |
| --- | --- | --- | --- | --- | --- |
|  | **Gram Negative** | **Gram Positive** | **Mixed** | **Sterile** | **Total** |
| **Gram Negative** | 104 | 2 | 0 | 12 | **118** |
| **Gram Positive** | 58 | 40 | 1 | 73 | **172** |
| **Mixed** | 121 | 5 | 5 | 12 | **143** |
| **Non-classifiable** | 12 | 4 | 1 | 72 | **89** |
| **No flag** | 23 | 8 | 0 | 168 | **199** |
| **Total** | **318** | **59** | **7** | **337** | **721** |
