# Supplementary material for: Multicenter evaluation of BACT-Info. and an infection algorithm using Urine Flow Cytometry among clinically diagnosed UTI patients in Indonesia
Source: PLoS One. 2026 Jul 15;21(7):e0339255. doi: 10.1371/journal.pone.0339255 (PMC13372243; doi:10.1371/journal.pone.0339255)
Supplement: S4 Table — UF-5000/4000 Flag Results Based on Presumptive Microscopic Gram Type of Urine. When the UF-5000/4000 system results were compared to the presumptive microscopic Gram typing, 86 out of 240 culture-confirmed Gram-negative cases were correctly flagged by the UF as Gram-negative, while the remaining cases were misclassified under different categories (table 11). Similarly, out of 117 presumptive microscopic Gram-positive cases, 53 were correctly identified by the UF system, and 64 were misclassified as various categories. Mixed and non-classifiable BACT-Info. flag results also showed varied distribution with the UF system identifying 24 mixed infections, consistent with the presumptive Gram results, and 70 non-classified results categorized as having no bacteria found. The “No flag” category in the UF system had the highest number of sterile cases (165 out of 199), indicating the system’s tendency not to flag when bacterial presence was low or absent. This category also showed the highest agreement with the absence of bacteria in presumptive Gram results. (DOCX) [file pone.0339255.s005.docx]

| **UF BACT-Info. Flag** | **Presumptive Gram Type** | | | | |
| --- | --- | --- | --- | --- | --- |
|  | **Gram Negative** | **Gram Positive** | **Mixed** | **No bacteria found** | **Total** |
| **Gram Negative** | 86 | 10 | 5 | 17 | **118** |
| **Gram Positive** | 52 | 53 | 10 | 57 | **172** |
| **Mixed** | 78 | 27 | 24 | 14 | **143** |
| **Non-classifiable** | 11 | 7 | 1 | 70 | **89** |
| **No flag** | 13 | 19 | 2 | 165 | **199** |
| **Total** | **240** | **117** | **42** | **323** | **721** |
