# Supplementary material for: Multicenter evaluation of BACT-Info. and an infection algorithm using Urine Flow Cytometry among clinically diagnosed UTI patients in Indonesia
Source: PLoS One. 2026 Jul 15;21(7):e0339255. doi: 10.1371/journal.pone.0339255 (PMC13372243; doi:10.1371/journal.pone.0339255)
Supplement: Attachment — Submitted filename: Response to reviewers - April 27.docx [file pone.0339255.s008.docx]

Dear Dr. Polilli,

We thank the Academic Editor and the reviewers for their careful evaluation of our manuscript entitled: **“**Multicenter Evaluation of BACT-Info. and an Infection Algorithm Using Urine Flow Cytometry among Clinically Diagnosed UTI Patients in Indonesia.”

Their constructive comments have helped us improve the methodological clarity, transparency, and overall quality of the manuscript. All comments have been addressed individually below, and corresponding revisions have been incorporated into the revised manuscript.

We appreciate the suggestion to deposit our laboratory protocols in protocols.io to further enhance reproducibility and transparency. In the current revision, we have focused on ensuring that all relevant methodological details are described as clearly and comprehensively as possible within the manuscript. We hope that the information provided will adequately support the reproducibility of our study, and we remain willing to provide additional clarification on specific aspects if required.

**Journal Requirements:**

1. Please ensure that your manuscript meets PLOS ONE's style requirements, including those for file naming. The PLOS ONE style templates can be found at

https://journals.plos.org/plosone/s/file?id=wjVg/PLOSOne_formatting_sample_main_body.pdf and

<https://journals.plos.org/plosone/s/file?id=ba62/PLOSOne_formatting_sample_title_authors_affiliations.pdf>.

**Response**

We thank the editor for this reminder. The manuscript has been carefully revised to comply with the formatting guidelines of PLOS ONE.

Author contributions were previously defined during submission; therefore, the corresponding statement has been removed from the revised manuscript to avoid redundancy

2. Thank you for stating in your Funding Statement:

“• Specific grant numbers: The funding for this study was supported by Sysmex Asia Pacific Private Limited Company with grant number: PEA0007/MKT/AZS/XII/2022P
• Initials of authors who received each award: A.D.; T.L.; A.; R.T.; M.R.; I.P.; D.L.; N.S.D.; Y.E.K.; M.I.D.P; B.R.A.S.; L.S.; F.R.M.; H.K.; A.N.; R.A.M.; A.D.; L.L.; S.H.; I N.W.; R.E.K.; I.P.; H.S.; A.I.
• Full names of commercial companies that funded the study or authors: Sysmex
• Initials of authors who received salary or other funding from commercial companies: none
• URLs to sponsors’ websites: https://www.sysmex-ap.com/; https://www.sysmex.co.id/?lang=id”

Please provide an amended statement that declares *all* the funding or sources of support (whether external or internal to your organization) received during this study, as detailed online in our guide for authors at http://journals.plos.org/plosone/s/submit-now.  Please also include the statement “There was no additional external funding received for this study.” in your updated Funding Statement.

Please include your amended Funding Statement within your cover letter. We will change the online submission form on your behalf.

**Funding Statement**

This study was supported by Sysmex Asia Pacific Private Limited Company (Grant No. PEA0007/MKT/AZS/XII/2022P). The funder provided financial support for the study but had no role in data collection and analysis and the decision to publish. The authors received no salaries or other financial benefits from the company related to this study. There was no additional external funding received for this study.

This revised statement has been included in the cover letter, as requested by the editor.

3. We note that Figure 1 in your submission contain [map/satellite] images which may be copyrighted. All PLOS content is published under the Creative Commons Attribution License (CC BY 4.0), which means that the manuscript, images, and Supporting Information files will be freely available online, and any third party is permitted to access, download, copy, distribute, and use these materials in any way, even commercially, with proper attribution. For these reasons, we cannot publish previously copyrighted maps or satellite images created using proprietary data, such as Google software (Google Maps, Street View, and Earth). For more information, see our copyright guidelines: http://journals.plos.org/plosone/s/licenses-and-copyright.

**Response**

Thank you for your careful review of our manuscript and for highlighting the concern regarding the figure.

In response, we have replaced the original figure with a new version that complies with the CC BY 4.0 license requirements. The updated figure has been included in the revised manuscript, with all necessary attributions clearly indicated.

Additionally, the elements used in the revised figure (Public Domain/CC0 1.0) meet the requirements for use under the CC BY 4.0 license (PLOS One), with details as follows:

1. Indonesia map – Source: <https://commons.wikimedia.org/wiki/File:Indonesia.svg>
2. Location pin – Source: <https://publicdomainvectors.org/en/free-clipart/Vector-image-of-black-location-pin/25495.html>
3. Font: Baskerville – Source: <https://en.wikipedia.org/wiki/Category:Public_domain_typefaces>
   This font is free to use under the Open Font License (<https://openfontlicense.org/open-font-license-official-text/>).

We hope this revision addresses your concern. Please let us know if any further adjustments are needed.

**Reviewers' comments:**

**Reviewer #1**: I think the authors of this manuscript have done a really good job.
I recommend that they have a closer look at some typos or grammatical errors. For example line 147: "...urine culture resulted..." maybe you meant results?

Response:

We thank the reviewer for the correction. The grammatical error has been fixed (Page 8, Line 163).

**Reviewer #2**: The subject is of great interest and the paper can be improved by performing the requested modifications.

Line 90: it would be helpful for the reader to explain what “time-consuming” means in the context of a urine culture.

Line 98: Is an automated urinalysis system using flow cytometry expensive? Could it be compared with conventional urine analysis in terms of cost?

Line 102: Please clearly describe “UTI-Info. and BACT-Info” to introduce these terms to the reader.

Line 171: remove the point and line after ‘accuracy”

Line 293: correct “cutoffs” to “cut-offs”

**Response:**

We thank the reviewer for the positive evaluation of our work and for the helpful suggestions to improve the manuscript. We have carefully revised the manuscript and addressed all comments as outlined below.

1. We have revised the text to clarify what is meant by “time-consuming,” specifically indicating the typical turnaround time required for urine culture processing and reporting (Page 5, Lines 91-93).
2. We thank the reviewer for this important question regarding cost considerations. A direct cost comparison between automated urinalysis systems using flow cytometry and conventional urine analysis was not within the scope of our study, and to our knowledge, such comparative data are limited in Indonesia. However, we have added a clarification in the manuscript acknowledging this limitation and noting that evidence from international studies exists. For example, a cost-effectiveness analysis from Spain suggests that automated urinalysis may improve efficiency and reduce overall laboratory workload, although cost structures may vary by healthcare system. We have cited this study in the revised manuscript (Page 5, Line 99-107).
3. We have revised the manuscript to provide a clearer description and definition of “UTI-Info.” and “BACT-Info” at their first mention (Page 6, Lines 112–113).
4. Regarding the point and line after “accuracy”, this has been corrected in the revised manuscript (Page 9, Line 187).
5. Regarding cutoffs, this has been corrected in the revised manuscript (Page 19, Line 304).

**Reviewer #3**: 1- The manuscript refers to an “infection algorithm,” but the description is insufficient for reproducibility. Please clearly explain how the algorithm works, including the thresholds, parameters used, and whether it was predefined by the manufacturer or developed within the study. Although the manuscript states that suspected UTI patients were recruited from multiple centers, the criteria used to define “suspected UTI” should be described more explicitly to ensure reproducibility and minimize selection bias. The manuscript should clearly explain how contaminated samples or mixed bacterial growth were classified and whether these samples were excluded from the final diagnostic accuracy analysis. Please clarify whether the investigators interpreting the UF-5000 results were blinded to urine culture results and vice versa. Proper blinding is essential to minimize diagnostic review bias.

Response:

We thank the reviewer for this important comment. We agree that a clearer description of the infection algorithm is necessary to ensure reproducibility.

Accordingly, we have revised the manuscript to provide a more detailed explanation of the algorithm, including the specific parameters used (nitrite test, leukocyturia, and urine flow cytometry flags such as “UTI-Info.” and “BACT-Info.”) and the corresponding decision pathways. We have expanded the description of the algorithm in the text to improve clarity and reproducibility (Page 26-28, Line 451-482).

The definition of clinically suspected UTIs has been added to the manuscript (Page 7, Line 136-140).

The performance of the BACT Info. and UTI Info. flags from the automated urinalysis system was evaluated against presumptive Gram staining and urine culture, both of which were performed independently and in a blinded manner. The Methods section has been revised to clarify this procedure and improve transparency and reproducibility. (Page 6, Line 127-129).

Regarding the samples with mixed bacterial growth, they were excluded from the final diagnostic accuracy analysis to ensure accurate evaluation of the urine flow cytometry (UF) performance. We have revised the Results section to explicitly state this, clarifying the classification and exclusion of mixed samples (Page 22, Line 363-367).

2-The statistical analysis section should include more detailed descriptions of how sensitivity, specificity, predictive values, and likelihood ratios were calculated, including the software used and the confidence intervals applied. While the UF-5000 system demonstrated high sensitivity, the reported specificity is relatively modest. The authors should provide a deeper discussion on the clinical implications of this finding, particularly the potential for false positives and unnecessary follow-up testing. If possible, the authors should present subgroup analyses based on patient characteristics (e.g., sex, inpatient vs. outpatient, age groups). Such analyses could improve understanding of the test's performance across different populations.

Response:

We thank the reviewer for this suggestion. We have revised the Statistical Analysis section to provide more detail. The details have been included in the revised manuscript to ensure clarity and reproducibility (Page 9, Lines 187-190).

We have added a discussion regarding the implications of modest specificity, emphasizing that the high sensitivity allows effective screening that may reduce unnecessary urine cultures and optimize antibiotic use (Page 26, Lines 433-435 and 439-443).

We thank the reviewer for the valuable suggestion on the subgroup analyses. We agree that the analyses based on patient characteristics could provide additional insights into test performance. However, the present study was not specifically designed to evaluate diagnostic performance across subgroups. In addition, some subgroups had limited sample sizes, which would further reduce the robustness of such comparisons.

Given these considerations, we focused on the overall diagnostic performance of the system, which reflects its intended use as a screening tool in routine clinical practice.

3- While the authors cite previous studies evaluating urine flow cytometry systems, the discussion would benefit from a more detailed comparison of diagnostic performance metrics with previously published research. The manuscript would be strengthened by outlining how this screening approach could be implemented in routine clinical laboratories, including potential benefits in reducing unnecessary urine cultures and improving laboratory efficiency.

Response:

We thank the reviewer for this valuable suggestion. We have revised the Discussion section to provide a more detailed comparison of our findings with previously published studies. We have expanded the discussion part to describe practical implementation of the UF-based screening approach in routine clinical laboratories. We outline how combining UF flags with standard urinalysis parameters can reduce unnecessary urine cultures, streamline workflow, and improve laboratory efficiency, particularly in high-throughput or resource-limited settings (Page 24, Lines 392-402).
