# Supplementary material for: Multicenter evaluation of BACT-Info. and an infection algorithm using Urine Flow Cytometry among clinically diagnosed UTI patients in Indonesia
Source: PLoS One. 2026 Jul 15;21(7):e0339255. doi: 10.1371/journal.pone.0339255 (PMC13372243; doi:10.1371/journal.pone.0339255)
Supplement: Attachment — Submitted filename: Minor revision.docx [file pone.0339255.s006.docx]

The subject is of great interest and the paper can be improved by performing the requested modifications.

Line 90: it would be helpful for the reader to explain what “time-consuming” means in the context of a urine culture.

Line 98: Is an automated urinalysis system using flow cytometry expensive? Could it be compared with conventional urine analysis in terms of cost?

Line 102: Please clearly describe “UTI-Info. and BACT-Info” to introduce these terms to the reader.

Line 171: remove the point and line after ‘accuracy”

Line 293: correct “cutoffs” to “cut-offs”
