# Supplementary material for: Multicenter evaluation of BACT-Info. and an infection algorithm using Urine Flow Cytometry among clinically diagnosed UTI patients in Indonesia
Source: PLoS One. 2026 Jul 15;21(7):e0339255. doi: 10.1371/journal.pone.0339255 (PMC13372243; doi:10.1371/journal.pone.0339255)
Supplement: Peer review history

# Peer review: Decision Letter 0

**DOI:** 10.1371/journal.pone.0339255.r001

**Authors:** Ennio Polilli

9 Mar 2026

PONE-D-25-63614

Multicenter Evaluation of BACT-Info. and an Infection Algorithm Using Urine Flow Cytometry among Clinically Diagnosed UTI Patients in Indonesia

PLOS One

Dear Dr. Dahesihdewi,

Thank you for submitting your manuscript to PLOS ONE. After careful consideration, we feel that it has merit but does not fully meet PLOS ONE’s publication criteria as it currently stands. Therefore, we invite you to submit a revised version of the manuscript that addresses the points raised during the review process.

Please submit your revised manuscript by Apr 23 2026 11:59PM. If you will need more time than this to complete your revisions, please reply to this message or contact the journal office at plosone@plos.org. When you're ready to submit your revision, log on to https://www.editorialmanager.com/pone/ and select the 'Submissions Needing Revision' folder to locate your manuscript file.

Please include the following items when submitting your revised manuscript:

- A letter that responds to each point raised by the academic editor and reviewer(s). You should upload this letter as a separate file labeled 'Response to Reviewers'.
- A marked-up copy of your manuscript that highlights changes made to the original version. You should upload this as a separate file labeled 'Revised Manuscript with Track Changes'.
- An unmarked version of your revised paper without tracked changes. You should upload this as a separate file labeled 'Manuscript'.

If you would like to make changes to your financial disclosure, please include your updated statement in your cover letter. Guidelines for resubmitting your figure files are available below the reviewer comments at the end of this letter.

If applicable, we recommend that you deposit your laboratory protocols in protocols.io to enhance the reproducibility of your results. Protocols.io assigns your protocol its own identifier (DOI) so that it can be cited independently in the future. For instructions see: https://journals.plos.org/plosone/s/submission-guidelines#loc-laboratory-protocols. Additionally, PLOS ONE offers an option for publishing peer-reviewed Lab Protocol articles, which describe protocols hosted on protocols.io. Read more information on sharing protocols at https://plos.org/protocols?utm_medium=editorial-email&utm_source=authorletters&utm_campaign=protocols.

We look forward to receiving your revised manuscript.

Kind regards,

Ennio Polilli

Academic Editor

PLOS One

Journal Requirements:

When submitting your revision, we need you to address these additional requirements.

1. Please ensure that your manuscript meets PLOS ONE's style requirements, including those for file naming. The PLOS ONE style templates can be found at

https://journals.plos.org/plosone/s/file?id=wjVg/PLOSOne_formatting_sample_main_body.pdf and

https://journals.plos.org/plosone/s/file?id=ba62/PLOSOne_formatting_sample_title_authors_affiliations.pdf.

2. Thank you for stating in your Funding Statement:

“• Specific grant numbers: The funding for this study was supported by Sysmex Asia Pacific Private Limited Company with grant number: PEA0007/MKT/AZS/XII/2022P

• Initials of authors who received each award: A.D.; T.L.; A.; R.T.; M.R.; I.P.; D.L.; N.S.D.; Y.E.K.; M.I.D.P; B.R.A.S.; L.S.; F.R.M.; H.K.; A.N.; R.A.M.; A.D.; L.L.; S.H.; I N.W.; R.E.K.; I.P.; H.S.; A.I.

• Full names of commercial companies that funded the study or authors: Sysmex

• Initials of authors who received salary or other funding from commercial companies: none

• URLs to sponsors’ websites: https://www.sysmex-ap.com/; https://www.sysmex.co.id/?lang=id”

Please provide an amended statement that declares *all* the funding or sources of support (whether external or internal to your organization) received during this study, as detailed online in our guide for authors at http://journals.plos.org/plosone/s/submit-now.  Please also include the statement “There was no additional external funding received for this study.” in your updated Funding Statement.

Please include your amended Funding Statement within your cover letter. We will change the online submission form on your behalf.

3. We note that Figure 1 in your submission contain [map/satellite] images which may be copyrighted. All PLOS content is published under the Creative Commons Attribution License (CC BY 4.0), which means that the manuscript, images, and Supporting Information files will be freely available online, and any third party is permitted to access, download, copy, distribute, and use these materials in any way, even commercially, with proper attribution. For these reasons, we cannot publish previously copyrighted maps or satellite images created using proprietary data, such as Google software (Google Maps, Street View, and Earth). For more information, see our copyright guidelines: http://journals.plos.org/plosone/s/licenses-and-copyright.

We require you to either (1) present written permission from the copyright holder to publish these figures specifically under the CC BY 4.0 license, or (2) remove the figures from your submission:

a. You may seek permission from the original copyright holder of Figure 1 to publish the content specifically under the CC BY 4.0 license.

We recommend that you contact the original copyright holder with the Content Permission Form (http://journals.plos.org/plosone/s/file?id=7c09/content-permission-form.pdf) and the following text:

“I request permission for the open-access journal PLOS ONE to publish XXX under the Creative Commons Attribution License (CCAL) CC BY 4.0 (http://creativecommons.org/licenses/by/4.0/). Please be aware that this license allows unrestricted use and distribution, even commercially, by third parties. Please reply and provide explicit written permission to publish XXX under a CC BY license and complete the attached form.”

Please upload the completed Content Permission Form or other proof of granted permissions as an "Other" file with your submission.

In the figure caption of the copyrighted figure, please include the following text: “Reprinted from [ref] under a CC BY license, with permission from [name of publisher], original copyright [original copyright year].”

b. If you are unable to obtain permission from the original copyright holder to publish these figures under the CC BY 4.0 license or if the copyright holder’s requirements are incompatible with the CC BY 4.0 license, please either i) remove the figure or ii) supply a replacement figure that complies with the CC BY 4.0 license. Please check copyright information on all replacement figures and update the figure caption with source information. If applicable, please specify in the figure caption text when a figure is similar but not identical to the original image and is therefore for illustrative purposes only.

The following resources for replacing copyrighted map figures may be helpful:

USGS National Map Viewer (public domain): http://viewer.nationalmap.gov/viewer/

The Gateway to Astronaut Photography of Earth (public domain): http://eol.jsc.nasa.gov/sseop/clickmap/

Maps at the CIA (public domain): https://www.cia.gov/library/publications/the-world-factbook/index.html and https://www.cia.gov/library/publications/cia-maps-publications/index.html

NASA Earth Observatory (public domain): http://earthobservatory.nasa.gov/

Landsat: http://landsat.visibleearth.nasa.gov/

USGS EROS (Earth Resources Observatory and Science (EROS) Center) (public domain): http://eros.usgs.gov/#

Natural Earth (public domain): http://www.naturalearthdata.com/

4. If the reviewer comments include a recommendation to cite specific previously published works, please review and evaluate these publications to determine whether they are relevant and should be cited. There is no requirement to cite these works unless the editor has indicated otherwise.

[Note: HTML markup is below. Please do not edit.]

Reviewers' comments:

Reviewer's Responses to Questions

**Comments to the Author**

1. Is the manuscript technically sound, and do the data support the conclusions?

The manuscript must describe a technically sound piece of scientific research with data that supports the conclusions. Experiments must have been conducted rigorously, with appropriate controls, replication, and sample sizes. The conclusions must be drawn appropriately based on the data presented.

Reviewer #1: Yes

Reviewer #2: Yes

Reviewer #3: Yes

**********

2. Has the statistical analysis been performed appropriately and rigorously?

Reviewer #1: Yes

Reviewer #2: Yes

Reviewer #3: Yes

**********

3. Have the authors made all data underlying the findings in their manuscript fully available?

The PLOS Data policy requires authors to make all data underlying the findings described in their manuscript fully available without restriction, with rare exception (please refer to the Data Availability Statement in the manuscript PDF file). The data should be provided as part of the manuscript or its supporting information, or deposited to a public repository. For example, in addition to summary statistics, the data points behind means, medians and variance measures should be available. If there are restrictions on publicly sharing data—e.g. participant privacy or use of data from a third party—those must be specified.

Reviewer #1: Yes

Reviewer #2: Yes

Reviewer #3: Yes

**********

4. Is the manuscript presented in an intelligible fashion and written in standard English?

PLOS ONE does not copyedit accepted manuscripts, so the language in submitted articles must be clear, correct, and unambiguous. Any typographical or grammatical errors should be corrected at revision, so please note any specific errors here.

Reviewer #1: Yes

Reviewer #2: Yes

Reviewer #3: Yes

**********

5. Review Comments to the Author

Please use the space provided to explain your answers to the questions above. You may also include additional comments for the author, including concerns about dual publication, research ethics, or publication ethics. (Please upload your review as an attachment if it exceeds 20,000 characters)

Reviewer #1: I think the authors of this manuscript have done a really good job.

I recommend that they have a closer look at some typos or grammatical errors. For example line 147: "...urine culture resulted..." maybe you meant results?

Reviewer #2: The subject is of great interest and the paper can be improved by performing the requested modifications.

Line 90: it would be helpful for the reader to explain what “time-consuming” means in the context of a urine culture.

Line 98: Is an automated urinalysis system using flow cytometry expensive? Could it be compared with conventional urine analysis in terms of cost?

Line 102: Please clearly describe “UTI-Info. and BACT-Info” to introduce these terms to the reader.

Line 171: remove the point and line after ‘accuracy”

Line 293: correct “cutoffs” to “cut-offs”

Reviewer #3: 1- The manuscript refers to an “infection algorithm,” but the description is insufficient for reproducibility. Please clearly explain how the algorithm works, including the thresholds, parameters used, and whether it was predefined by the manufacturer or developed within the study. Although the manuscript states that suspected UTI patients were recruited from multiple centers, the criteria used to define “suspected UTI” should be described more explicitly to ensure reproducibility and minimize selection bias. The manuscript should clearly explain how contaminated samples or mixed bacterial growth were classified and whether these samples were excluded from the final diagnostic accuracy analysis. Please clarify whether the investigators interpreting the UF-5000 results were blinded to urine culture results and vice versa. Proper blinding is essential to minimize diagnostic review bias.

2-The statistical analysis section should include more detailed descriptions of how sensitivity, specificity, predictive values, and likelihood ratios were calculated, including the software used and the confidence intervals applied. While the UF-5000 system demonstrated high sensitivity, the reported specificity is relatively modest. The authors should provide a deeper discussion on the clinical implications of this finding, particularly the potential for false positives and unnecessary follow-up testing. If possible, the authors should present subgroup analyses based on patient characteristics (e.g., sex, inpatient vs. outpatient, age groups). Such analyses could improve understanding of the test's performance across different populations.

3- While the authors cite previous studies evaluating urine flow cytometry systems, the discussion would benefit from a more detailed comparison of diagnostic performance metrics with previously published research. The manuscript would be strengthened by outlining how this screening approach could be implemented in routine clinical laboratories, including potential benefits in reducing unnecessary urine cultures and improving laboratory efficiency.

**********

6. PLOS authors have the option to publish the peer review history of their article (what does this mean?). If published, this will include your full peer review and any attached files.

If you choose “no”, your identity will remain anonymous but your review may still be made public.

**Do you want your identity to be public for this peer review?**  For information about this choice, including consent withdrawal, please see our Privacy Policy.

Reviewer #1: No

Reviewer #2: **Yes:** Mihaela Anca DASCALU

Reviewer #3: **Yes:** HAMZA ISLAM

**********

[NOTE: If reviewer comments were submitted as an attachment file, they will be attached to this email and accessible via the submission site. Please log into your account, locate the manuscript record, and check for the action link "View Attachments". If this link does not appear, there are no attachment files.]

To ensure your figures meet our technical requirements, please review our figure guidelines: https://journals.plos.org/plosone/s/figures

You may also use PLOS’s free figure tool, NAAS, to help you prepare publication quality figures: https://journals.plos.org/plosone/s/figures#loc-tools-for-figure-preparation.

NAAS will assess whether your figures meet our technical requirements by comparing each figure against our figure specifications.

Attachment Submitted filename: Minor revision.docx

# Author comment: Author response to Decision Letter 1

**DOI:** 10.1371/journal.pone.0339255.r002

27 Apr 2026

Dear Dr. Polilli,

We thank the Academic Editor and the reviewers for their careful evaluation of our manuscript entitled: “Multicenter Evaluation of BACT-Info. and an Infection Algorithm Using Urine Flow Cytometry among Clinically Diagnosed UTI Patients in Indonesia.”

Their constructive comments have helped us improve the methodological clarity, transparency, and overall quality of the manuscript. All comments have been addressed individually below, and corresponding revisions have been incorporated into the revised manuscript.

We appreciate the suggestion to deposit our laboratory protocols in protocols.io to further enhance reproducibility and transparency. In the current revision, we have focused on ensuring that all relevant methodological details are described as clearly and comprehensively as possible within the manuscript. We hope that the information provided will adequately support the reproducibility of our study, and we remain willing to provide additional clarification on specific aspects if required.

Journal Requirements:

1. Please ensure that your manuscript meets PLOS ONE's style requirements, including those for file naming. The PLOS ONE style templates can be found at

https://journals.plos.org/plosone/s/file?id=wjVg/PLOSOne_formatting_sample_main_body.pdf and

https://journals.plos.org/plosone/s/file?id=ba62/PLOSOne_formatting_sample_title_authors_affiliations.pdf.

Response

We thank the editor for this reminder. The manuscript has been carefully revised to comply with the formatting guidelines of PLOS ONE.

Author contributions were previously defined during submission; therefore, the corresponding statement has been removed from the revised manuscript to avoid redundancy

2. Thank you for stating in your Funding Statement:

“• Specific grant numbers: The funding for this study was supported by Sysmex Asia Pacific Private Limited Company with grant number: PEA0007/MKT/AZS/XII/2022P

• Initials of authors who received each award: A.D.; T.L.; A.; R.T.; M.R.; I.P.; D.L.; N.S.D.; Y.E.K.; M.I.D.P; B.R.A.S.; L.S.; F.R.M.; H.K.; A.N.; R.A.M.; A.D.; L.L.; S.H.; I N.W.; R.E.K.; I.P.; H.S.; A.I.

• Full names of commercial companies that funded the study or authors: Sysmex

• Initials of authors who received salary or other funding from commercial companies: none

• URLs to sponsors’ websites: https://www.sysmex-ap.com/; https://www.sysmex.co.id/?lang=id”

Please provide an amended statement that declares *all* the funding or sources of support (whether external or internal to your organization) received during this study, as detailed online in our guide for authors at http://journals.plos.org/plosone/s/submit-now. Please also include the statement “There was no additional external funding received for this study.” in your updated Funding Statement.

Please include your amended Funding Statement within your cover letter. We will change the online submission form on your behalf.

Funding Statement

This study was supported by Sysmex Asia Pacific Private Limited Company (Grant No. PEA0007/MKT/AZS/XII/2022P). The funder provided financial support for the study but had no role in data collection and analysis and the decision to publish. The authors received no salaries or other financial benefits from the company related to this study. There was no additional external funding received for this study.

This revised statement has been included in the cover letter, as requested by the editor.

3. We note that Figure 1 in your submission contain [map/satellite] images which may be copyrighted. All PLOS content is published under the Creative Commons Attribution License (CC BY 4.0), which means that the manuscript, images, and Supporting Information files will be freely available online, and any third party is permitted to access, download, copy, distribute, and use these materials in any way, even commercially, with proper attribution. For these reasons, we cannot publish previously copyrighted maps or satellite images created using proprietary data, such as Google software (Google Maps, Street View, and Earth). For more information, see our copyright guidelines: http://journals.plos.org/plosone/s/licenses-and-copyright.

Response

We thank the editorial team for highlighting copyright concerns. Figure 1 was created using free-to-use images from Vecteezy (“Indonesia Map PNGs”) and Google Maps icons by Smashicons from Flaticon. These resources are licensed for free use with proper attribution.

Reviewers' comments:

Reviewer #1: I think the authors of this manuscript have done a really good job.

I recommend that they have a closer look at some typos or grammatical errors. For example line 147: "...urine culture resulted..." maybe you meant results?

Response:

We thank the reviewer for the correction. The grammatical error has been fixed (Page 8, Line 163).

Reviewer #2: The subject is of great interest and the paper can be improved by performing the requested modifications.

Line 90: it would be helpful for the reader to explain what “time-consuming” means in the context of a urine culture.

Line 98: Is an automated urinalysis system using flow cytometry expensive? Could it be compared with conventional urine analysis in terms of cost?

Line 102: Please clearly describe “UTI-Info. and BACT-Info” to introduce these terms to the reader.

Line 171: remove the point and line after ‘accuracy”

Line 293: correct “cutoffs” to “cut-offs”

Response:

We thank the reviewer for the positive evaluation of our work and for the helpful suggestions to improve the manuscript. We have carefully revised the manuscript and addressed all comments as outlined below.

1. We have revised the text to clarify what is meant by “time-consuming,” specifically indicating the typical turnaround time required for urine culture processing and reporting (Page 5, Lines 91-93).

2. We thank the reviewer for this important question regarding cost considerations. A direct cost comparison between automated urinalysis systems using flow cytometry and conventional urine analysis was not within the scope of our study, and to our knowledge, such comparative data are limited in Indonesia. However, we have added a clarification in the manuscript acknowledging this limitation and noting that evidence from international studies exists. For example, a cost-effectiveness analysis from Spain suggests that automated urinalysis may improve efficiency and reduce overall laboratory workload, although cost structures may vary by healthcare system. We have cited this study in the revised manuscript (Page 5, Line 99-107).

3. We have revised the manuscript to provide a clearer description and definition of “UTI-Info.” and “BACT-Info” at their first mention (Page 6, Lines 112–113).

4. Regarding the point and line after “accuracy”, this has been corrected in the revised manuscript (Page 9, Line 187).

5. Regarding cutoffs, this has been corrected in the revised manuscript (Page 19, Line 304).

Reviewer #3: 1- The manuscript refers to an “infection algorithm,” but the description is insufficient for reproducibility. Please clearly explain how the algorithm works, including the thresholds, parameters used, and whether it was predefined by the manufacturer or developed within the study. Although the manuscript states that suspected UTI patients were recruited from multiple centers, the criteria used to define “suspected UTI” should be described more explicitly to ensure reproducibility and minimize selection bias. The manuscript should clearly explain how contaminated samples or mixed bacterial growth were classified and whether these samples were excluded from the final diagnostic accuracy analysis. Please clarify whether the investigators interpreting the UF-5000 results were blinded to urine culture results and vice versa. Proper blinding is essential to minimize diagnostic review bias.

Response:

We thank the reviewer for this important comment. We agree that a clearer description of the infection algorithm is necessary to ensure reproducibility.

Accordingly, we have revised the manuscript to provide a more detailed explanation of the algorithm, including the specific parameters used (nitrite test, leukocyturia, and urine flow cytometry flags such as “UTI-Info.” and “BACT-Info.”) and the corresponding decision pathways. We have expanded the description of the algorithm in the text to improve clarity and reproducibility (Page 26-28, Line 451-482).

The definition of clinically suspected UTIs has been added to the manuscript (Page 7, Line 136-140).

The performance of the BACT Info. and UTI Info. flags from the automated urinalysis system was evaluated against presumptive Gram staining and urine culture, both of which were performed independently and in a blinded manner. The Methods section has been revised to clarify this procedure and improve transparency and reproducibility. (Page 6, Line 127-129).

Regarding the samples with mixed bacterial growth, they were excluded from the final diagnostic accuracy analysis to ensure accurate evaluation of the urine flow cytometry (UF) performance. We have revised the Results section to explicitly state this, clarifying the classification and exclusion of mixed samples (Page 22, Line 363-367).

2-The statistical analysis section should include more detailed descriptions of how sensitivity, specificity, predictive values, and likelihood ratios were calculated, including the software used and the confidence intervals applied. While the UF-5000 system demonstrated high sensitivity, the reported specificity is relatively modest. The authors should provide a deeper discussion on the clinical implications of this finding, particularly the potential for false positives and unnecessary follow-up testing. If possible, the authors should present subgroup analyses based on patient characteristics (e.g., sex, inpatient vs. outpatient, age groups). Such analyses could improve understanding of the test's performance across different populations.

Response:

We thank the reviewer for this suggestion. We have revised the Statistical Analysis section to provide more detail. The details have been included in the revised manuscript to ensure clarity and reproducibility (Page 9, Lines 187-190).

We have added a discussion regarding the implications of modest specificity, emphasizing that the high sensitivity allows effective screening that may reduce unnecessary urine cultures and optimize antibiotic use (Page 26, Lines 433-435 and 439-443).

We thank the reviewer for the valuable suggestion on the subgroup analyses. We agree that the analyses based on patient characteristics could provide additional insights into test performance. However, the present study was not specifically designed to evaluate diagnostic performance across subgroups. In addition, some subgroups had limited sample sizes, which would further reduce the robustness of such comparisons.

Given these considerations, we focused on the overall diagnostic performance of the system, which reflects its intended use as a screening tool in routine clinical practice.

3- While the authors cite previous studies evaluating urine flow cytometry systems, the discussion would benefit from a more detailed comparison of diagnostic performance metrics with previously published research. The manuscript would be strengthened by outlining how this screening approach could be implemented in routine clinical laboratories, including potential benefits in reducing unnecessary urine cultures and improving laboratory efficiency.

Response:

We thank the reviewer for this valuable suggestion. We have revised the Discussion section to provide a more detailed comparison of our findings with previously published studies. We have expanded the discussion part to describe practical implementation of the UF-based screening approach in routine clinical laboratories. We outline how combining UF flags with standard urinalysis parameters can reduce unnecessary urine cultures, streamline workflow, and improve laboratory efficiency, particularly in high-throughput or resource-limited settings (Page 24, Lines 392-402).

Attachment Submitted filename: Response to reviewers - April 27.docx

# Peer review: Decision Letter 1

**DOI:** 10.1371/journal.pone.0339255.r003

**Authors:** Ennio Polilli

18 Jun 2026

Multicenter Evaluation of BACT-Info. and an Infection Algorithm Using Urine Flow Cytometry among Clinically Diagnosed UTI Patients in Indonesia

PONE-D-25-63614R1

Dear Dr. Dahesihdewi,

We’re pleased to inform you that your manuscript has been judged scientifically suitable for publication and will be formally accepted for publication once it meets all outstanding technical requirements.

Within one week, you’ll receive an e-mail detailing the required amendments. When these have been addressed, you’ll receive a formal acceptance letter and your manuscript will be scheduled for publication.

An invoice will be generated when your article is formally accepted. Please note, if your institution has a publishing partnership with PLOS and your article meets the relevant criteria, all or part of your publication costs will be covered. Please make sure your user information is up-to-date by logging into Editorial Manager at Editorial Manager® and clicking the ‘Update My Information' link at the top of the page. For questions related to billing, please contact billing support.

If your institution or institutions have a press office, please notify them about your upcoming paper to help maximize its impact. If they’ll be preparing press materials, please inform our press team as soon as possible -- no later than 48 hours after receiving the formal acceptance. Your manuscript will remain under strict press embargo until 2 pm Eastern Time on the date of publication. For more information, please contact onepress@plos.org.

Kind regards,

Ennio Polilli

Academic Editor

PLOS One

Additional Editor Comments (optional):

Reviewers' comments:

Reviewer's Responses to Questions

**Comments to the Author**

1. If the authors have adequately addressed your comments raised in a previous round of review and you feel that this manuscript is now acceptable for publication, you may indicate that here to bypass the “Comments to the Author” section, enter your conflict of interest statement in the “Confidential to Editor” section, and submit your "Accept" recommendation.

Reviewer #2: All comments have been addressed

Reviewer #3: All comments have been addressed

**********

2. Is the manuscript technically sound, and do the data support the conclusions?

The manuscript must describe a technically sound piece of scientific research with data that supports the conclusions. Experiments must have been conducted rigorously, with appropriate controls, replication, and sample sizes. The conclusions must be drawn appropriately based on the data presented.

Reviewer #2: Yes

Reviewer #3: Yes

**********

3. Has the statistical analysis been performed appropriately and rigorously?

Reviewer #2: Yes

Reviewer #3: Yes

**********

4. Have the authors made all data underlying the findings in their manuscript fully available?

The PLOS Data policy requires authors to make all data underlying the findings described in their manuscript fully available without restriction, with rare exception (please refer to the Data Availability Statement in the manuscript PDF file). The data should be provided as part of the manuscript or its supporting information, or deposited to a public repository. For example, in addition to summary statistics, the data points behind means, medians and variance measures should be available. If there are restrictions on publicly sharing data—e.g. participant privacy or use of data from a third party—those must be specified.

Reviewer #2: Yes

Reviewer #3: Yes

**********

5. Is the manuscript presented in an intelligible fashion and written in standard English?

PLOS ONE does not copyedit accepted manuscripts, so the language in submitted articles must be clear, correct, and unambiguous. Any typographical or grammatical errors should be corrected at revision, so please note any specific errors here.

Reviewer #2: Yes

Reviewer #3: Yes

**********

6. Review Comments to the Author

Please use the space provided to explain your answers to the questions above. You may also include additional comments for the author, including concerns about dual publication, research ethics, or publication ethics. (Please upload your review as an attachment if it exceeds 20,000 characters)

Reviewer #2: (No Response)

Reviewer #3: This version has satisfied my comments. Good job done. Good Luck.

All my comments related to statistiscal method are done accurately. Similarly the discussion is modified and I think this revised version should be published.

**********

7. PLOS authors have the option to publish the peer review history of their article (what does this mean?). If published, this will include your full peer review and any attached files.

If you choose “no”, your identity will remain anonymous but your review may still be made public.

**Do you want your identity to be public for this peer review?**  For information about this choice, including consent withdrawal, please see our Privacy Policy.

Reviewer #2: **Yes:** Mihaela Anca DASCALU

Reviewer #3: **Yes:** Hamza Islam

**********

# Editor report: Acceptance letter

**DOI:** 10.1371/journal.pone.0339255.r004

**Authors:** Ennio Polilli

PONE-D-25-63614R1

PLOS One

Dear Dr. Dahesihdewi,

I'm pleased to inform you that your manuscript has been deemed suitable for publication in PLOS One. Congratulations! Your manuscript is now being handed over to our production team.

At this stage, our production department will prepare your paper for publication. This includes ensuring the following:

* All references, tables, and figures are properly cited

* All relevant supporting information is included in the manuscript submission,

* There are no issues that prevent the paper from being properly typeset

You will receive further instructions from the production team, including instructions on how to review your proof when it is ready. Please keep in mind that we are working through a large volume of accepted articles, so please give us a few days to review your paper and let you know the next and final steps.

Lastly, if your institution or institutions have a press office, please let them know about your upcoming paper now to help maximize its impact. If they'll be preparing press materials, please inform our press team within the next 48 hours. Your manuscript will remain under strict press embargo until 2 pm Eastern Time on the date of publication. For more information, please contact onepress@plos.org.

You will receive an invoice from PLOS for your publication fee after your manuscript has reached the completed accept phase. If you receive an email requesting payment before acceptance or for any other service, this may be a phishing scheme. Learn how to identify phishing emails and protect your accounts at https://explore.plos.org/phishing.

If we can help with anything else, please email us at customercare@plos.org.

Thank you for submitting your work to PLOS ONE and supporting open access.

Kind regards,

PLOS ONE Editorial Office Staff

on behalf of

Dr. Ennio Polilli

Academic Editor

PLOS One
